# Assessing the Role of DNA Methylation-Derived Neutrophil-to-Lymphocyte Ratio in Rheumatoid Arthritis

**DOI:** 10.1155/2018/2624981

**Published:** 2018-08-14

**Authors:** Srikant Ambatipudi, Gemma C. Sharp, Sarah L. N. Clarke, Darren Plant, Jonathan H. Tobias, David M. Evans, Anne Barton, Caroline L. Relton

**Affiliations:** ^1^MRC Integrative Epidemiology Unit, Bristol Medical School, University of Bristol, Bristol, UK; ^2^Department of Paediatric Rheumatology, Bristol Royal Hospital for Children, Bristol, UK; ^3^NIHR Manchester Musculoskeletal Biomedical Research Unit, Manchester Academy of Health Sciences, Central Manchester NHS Trust, and Arthritis Research UK Centre for Genetics and Genomics, The University of Manchester, Manchester, UK; ^4^Musculoskeletal Research Unit, Bristol Medical School, University of Bristol, Bristol, UK; ^5^University of Queensland Diamantina Institute, Translational Research Institute, Brisbane, QLD, Australia

## Abstract

Rheumatoid arthritis (RA) is a disease of chronic systemic inflammation (SI). In the present study, we used four datasets to explore whether methylation-derived neutrophil-to-lymphocyte ratio (mdNLR) might be a marker of SI in new onset, untreated, and treated prevalent RA cases and/or a marker of treatment response to the tumour necrosis factor inhibitor (TNFi) etanercept. mdNLR was associated with increased odds of being a new onset RA case (OR = 2.32, 95% CI = 1.95–2.80, *P* < 2 × 10^−16^) and performed better in distinguishing new onset RA cases from controls compared to covariates: age, gender, and smoking status. In untreated preclinical RA cases and controls, mdNLR at baseline was associated with diagnosis of RA in later life after adjusting for batch (OR = 4.30, 95% CI = 1.52–21.71, *P* = 0.029) although no association was observed before batch correction. When prevalent RA cases were treated, there was no association with mdNLR in samples before and after batch correction (OR = 0.34, 95% CI = 0.05–1.82, *P* = 0.23), and mdNLR was not associated with treatment response to etanercept (OR = 1.10, 95% CI = 0.75–1.68, *P* = 0.64). Our results indicate that SI measured by DNA methylation data is indicative of the recent onset of RA. Although preclinical RA was associated with mdNLR, there was no difference in the mean mdNLR between preclinical RA cases and controls. mdNLR was not associated with RA case status if treatment for RA has commenced, and it is not associated with treatment response. In the future, mdNLR estimates may be used as a valuable research tool to reliably estimate SI in the absence of freshly collected blood samples.

## 1. Introduction

Rheumatoid arthritis (RA) is the most common inflammatory arthropathy, characterized by chronic systemic inflammation (SI) [1]. The pathophysiology of RA involves a complex interplay between different cells including leukocytes, synovial fibroblasts, chondrocytes, and osteoclasts that leads to loss of immune homeostasis [1]. Of all the cells implicated in the pathophysiology of RA, neutrophils possess the greatest cytotoxic potential owing to their ability to release degradative enzymes and reactive oxygen species [2]. They are activated by exposure to immune complexes, rheumatoid factors, and cytokines in synovial fluid [3]. In addition, the neutrophils interact with macrophages, dendritic cells (DCs), natural killer cells, mesenchymal stem cells, and lymphocytes influencing innate and adaptive immune responses leading to SI [3, 4]. Circulating blood cell components such as white blood cell and mean platelet volume are considered putative biomarkers of inflammatory activity [5, 6]. Clinically, this inflammatory activity can be measured by acute phase proteins [7], although recent studies have shown that aberrant neutrophil-to-lymphocyte ratio (NLR) may be used as a marker of SI in the development of coronary heart diseases [8, 9], solid tumours [10], and autoimmune diseases like Takayasu's arteritis [11] and RA [12]. Under certain circumstances such as anti-IL-6 therapy when C-reactive protein (CRP) levels are less useful in monitoring inflammation, NLR has been shown to be a better marker of evaluating disease activity in patients with RA [13]. However, leukocyte measures are not readily available in many studies, especially from prospective population-based cohorts due to archiving of blood samples. This limits the evaluation of immune parameters and immunomodulation which are of immense importance in a chronic disease research setting [14]. Two recent studies have shown the utility of a methylation-derived NLR (mdNLR) index from peripheral blood DNA as an alternative measure of NLR and have applied this as a marker of cancer development and progression [15, 16].

We hypothesized that SI in RA could be assessed by measuring mdNLR and could be used as a research tool for assessing SI especially in a chronic disease setting without the need for fresh samples. Further, we hypothesized that treatment for RA might reduce any association between RA and mdNLR. In this regard, we tested if mdNLR might be associated with treatment response to the tumour necrosis factor inhibitor (TNFi) etanercept.

## 2. Materials and Methods

**Figure 1:**
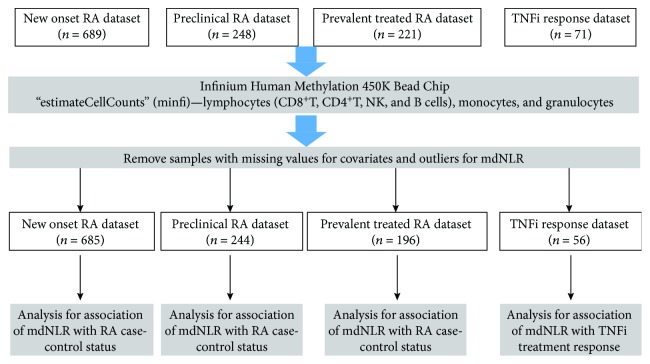
An overview of the study design.

Figure 1 provides an outline of the study design.

### 2.1. Study Samples

#### 2.1.1. New Onset RA Dataset

The raw methylation data and covariates for rheumatoid arthritis cases and controls were obtained from the publicly available Gene Expression Omnibus submitted dataset GSE42861 which was part of the Epidemiological Investigation of Rheumatoid Arthritis (EIRA) study [17, 18]. Only incident RA cases were invited for the study within the years 1996–2009 from middle Sweden. The controls matched by sex, age, smoking status, and residence area were selected from the same population as previously described [17]. Cells for isolating DNA were obtained from the patients during their first visit to the rheumatology department before giving any disease-modifying antirheumatic drugs (DMARDs) [19]. Methylation data were generated on DNA isolated from EDTA-treated blood samples of anticitrullinated protein antibody- (ACPA-) associated subtype of rheumatoid arthritis and controls using the Illumina HumanMethylation450 BeadChip array [17]. In the new onset RA dataset, of the 689 samples available for analysis (controls = 335, cases = 354), we removed 2 outlier samples (for mdNLR) and 2 samples with no information on smoking status, leaving us with 685 samples for the final analysis including 352 ACPA-associated RA cases and 333 controls.

#### 2.1.2. Preclinical RA Dataset

To assess whether mdNLR can be used as a predictive biomarker of future RA diagnosis in individuals who were disease-free at the time of blood collection, we used data from the Avon Longitudinal Study of Parents and Children (ALSPAC). DNA samples were collected when the women in the study were pregnant, which was 18 years prior to completing a questionnaire ascertaining RA status. The women were asked whether they had ever been diagnosed with RA by a doctor and what year they had first been diagnosed. HumanMethylation 450K BeadChip array data for roughly 1000 women were generated as part of the Accessible Resource for Integrated Epigenomics Studies (ARIES) project [20]. Of these, a random sample of 200 women who had never been diagnosed with any form of arthritis 18 years after enrolment were classified as “disease-free” controls for the purposes of the current study. A further 48 women were selected as “preclinical RA” cases (14 from the original ARIES study plus 34 ALSPAC women who had contributed a blood DNA sample during pregnancy but had not been included in ARIES). These women did not have a diagnosis of RA during pregnancy but received a diagnosis of RA later in life. Samples with missing values for covariates age and smoking status were removed, leaving 46 preclinical RA cases and 198 controls for the final analysis.

#### 2.1.3. Prevalent Treated RA Dataset

ALSPAC women also provided a blood sample at a follow-up clinic 18 years after pregnancy, around the same time that they completed the questions on RA diagnosis. HumanMethylation 450K data were available for 21 women with prevalent RA at this time point (15 from the original ARIES project plus 6 women who were not included in ARIES). All prevalent RA cases in this sample were assumed to be undergoing treatment for RA. A random sample of 200 controls (defined as women who reported that they had never been diagnosed with any form of arthritis) was also selected from the ARIES subsample. Samples with missing values for covariates age and smoking status were removed, leaving 20 prevalent RA cases and 176 controls for the final analysis.

#### 2.1.4. Treatment Response Dataset

To assess whether mdNLR can be used as a predictive biomarker of response to the tumour necrosis factor inhibitor (TNFi) etanercept in RA, we used the Biologics in Rheumatoid Arthritis Genetics and Genomics Study Syndicate (BRAGGSS) dataset, a prospective longitudinal study of response to biologic therapies in patients with RA. Illumina HumanMethylation 450K BeadChip array was used to generate DNA methylation data from pretreatment whole blood samples [21]. The DNA methylation dataset consisted of 36 very good responders (i.e., with clinical remission of their disease) and 35 nonresponders. Efficacy to TNFi was determined following 6 months on drug using established EULAR response criteria. The sample selection and preparation have been described previously [21]. Fifteen samples with missing information on smoking status were removed from the main analysis leaving 56 samples for the final analysis.

### 2.2. Data Preprocessing and Estimating Proportion of Leukocyte Cell Types

#### 2.2.1. New Onset RA Dataset

The raw data was normalized using subset-quantile within array normalization (SWAN) algorithm [22]. We removed bad quality probes (detection *P* > 0.01), probes containing SNPs in the CpG interrogation site or single-nucleotide extension site, cross-reactive probes, and probes on chromosomes X and Y [23]. Proportions of leukocyte subtypes—granulocytes, monocytes, and lymphocytes (CD4^+^T cells, CD8^+^T cells, B cells, and NK cells)—were estimated by (i) the “estimateCellCounts” function implemented in the Bioconductor package minfi [24] according to the Houseman method [25] and (ii) an optimized reference-based cell mixture deconvolution methodology IDOL [26]. Methylation-derived neutrophil-to-lymphocyte ratio (mdNLR) was estimated by dividing estimated proportions of granulocytes by lymphocytes as previously described [15]. Previous studies have reported a strong agreement between mdNLR and cytological NLR estimates instilling confidence in the DNA methylation-based estimates of leukocytes and methylation-derived SI [15, 16].

#### 2.2.2. Preclinical RA and Prevalent Treated RA Datasets

Peripheral blood samples were collected according to standard procedures, spun, and frozen at −80°C. Isolated DNA was bisulphite converted using the Zymo EZ DNA Methylation™ kit (Zymo, Irvine, CA). Following conversion, the genome-wide methylation status of over 485,000 CpG sites was measured using the Illumina HumanMethylation 450K BeadChip assay according to the standard protocol. The arrays were scanned using an Illumina iScan, and initial quality review was assessed using GenomeStudio (version 2011.1). Quality control of ARIES samples has been described previously [15]. Data for both time points (during pregnancy and 18 years after) and both subsamples (ARIES and extra samples from ALSPAC) were preprocessed as a single set in R (version 3.0.1) with the wateRmelon package according to the subset-quantile normalization approach [22]. The mdNLR was calculated as described above using the “estimateCellCounts” generated using the Houseman method in minfi [24, 25]. Data for the prevalent treated RA dataset and the preclinical RA dataset were normalized together.

#### 2.2.3. Treatment Response Dataset

Seventy-one samples were available for the assessment of mdNLR as a biomarker of response to TNFi (etanercept) therapy. The raw data were preprocessed and normalized as previously described [21]. The proportion of leukocyte subtypes was estimated by “estimateCellCounts” function implemented in minfi, and mdNLR was derived as described above.

### 2.3. Statistical Analyses

The analyses were performed using the statistical software R (version 3.4.0). Univariate and multivariate logistic regression was performed to test the association between mdNLR and RA status. A Wilcoxon rank sum test was performed to test if RA cases and controls differed in the levels of DNA methylation at five sites that have recently been identified as CpGs arising during myeloid differentiation that could serve as surrogates for mdNLR [16].

In the new onset RA dataset, Liu et al. reported an imbalance between the number of cases and controls run per date which may potentially confound our analysis. Surrogate variable analysis is used for identifying, estimating, and incorporating sources of variation in gene expression and DNA methylation analysis [27, 28]. To identify technical sources of variation, we performed SVA and derived ten surrogate variables in the new onset dataset. Individuals' age, gender, and smoking status have been previously shown to be associated with the risk of developing RA [29, 30]. Hence, we incorporated age, gender, and smoking status along with 10 surrogate variables in the statistical model. The ability of mdNLR to classify RA cases from controls was assessed using Receiver Operating Characteristic (ROC) curves and the corresponding area under the ROC curve (AUC) values using the R package pROC [31]. For the preclinical and prevalent treated RA datasets, multivariate logistic regression was performed to test the association between mdNLR and RA status (either current or future).

The statistical model was adjusted for individuals' age, smoking status, and bisulphite conversion plate. In the treatment response dataset, the association between mdNLR at the beginning of the treatment and response at 3 months was evaluated using a multivariate logistic regression model adjusting for individuals' age, gender, smoking status, use of DMARDs, and disease activity score 28.

## 3. Results

### 3.1. Sample Characteristics

The sample characteristics including demographic and epidemiological data for all four datasets are shown in Table 1. New onset RA, preclinical, and treated prevalent RA cases did not differ from controls in terms of mean age and smoking status (Table 1). In the treatment response dataset, the nonresponders were older and had a higher health assessment questionnaire score compared to good responders (Table 1).

### 3.2. mdNLR Is Elevated at Rheumatoid Arthritis Disease Onset

In the new onset RA dataset, we compared the mdNLR derived using two different algorithms (“estimateCellCounts” and IDOL) and found that the two methods of estimating mdNLR were highly correlated (*r* = 0.87, *P* < 2 × 10^−16^, Supplementary Figure 1). For this reason, the mdNLR derived using “estimateCellCounts” was used for further analyses.

**Figure 2:**
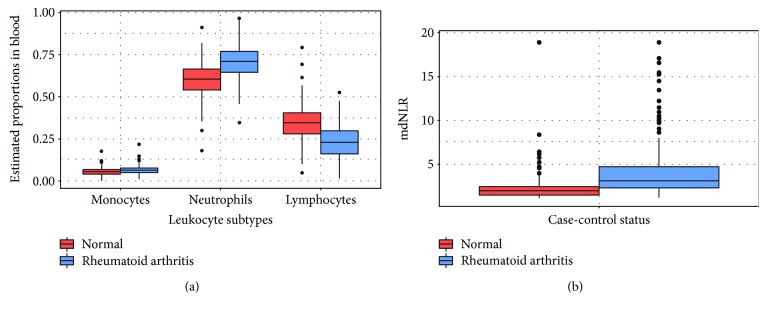
Leukocyte cell subtypes and mdNLR in new onset RA cases and controls. (a) Estimated proportions of leukocyte subtypes (monocytes, neutrophils, and lymphocytes) in controls and new onset RA cases. (b) Higher mdNLR SI index in new onset RA cases compared to controls (*P* < 2.2*e* − 16).

**Figure 3:**
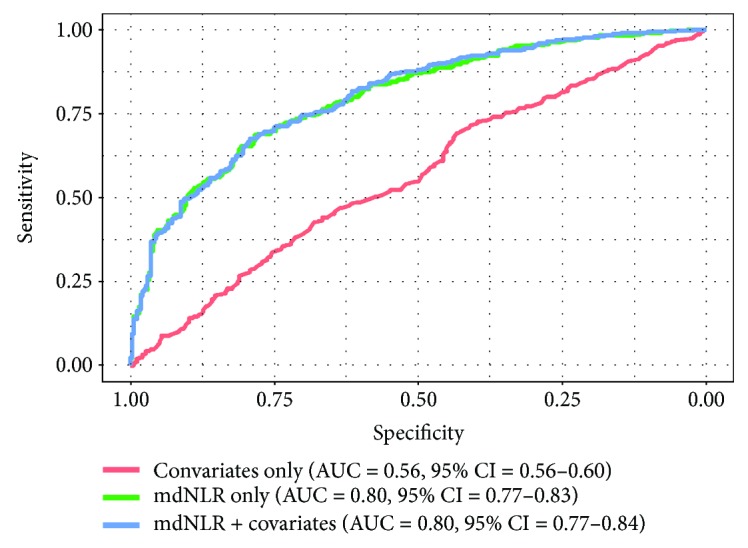
Diagnostic ability of mdNLR to distinguish new onset RA cases and controls. Each ROC curve was generated from a different classifier: shown below the figure with the area under the ROC curve (AUC) values. Covariates included age, gender, and smoking status.

We observed an elevated neutrophil and decreased lymphocyte count in new onset RA patients compared to controls (Figure 2(a)). The mean mdNLR for controls was 2.0, compared to 4.7 in new onset RA cases (Figure 2(b)). In a multivariate logistic regression model, a higher mdNLR index was associated with increased odds of being an RA case (OR = 2.32, 95% CI = 1.95–2.80, *P* < 2 × 10^−16^) as shown in Table 2. Further, we found that all five CpGs associated with myeloid differentiation (suggested to be a surrogate for mdNLR) were hypomethylated in RA cases compared to controls (Supplementary Table 1, Supplementary Figure 2). On its own, mdNLR was able to distinguish RA cases from controls with an AUC of 0.80 (95% CI = 0.77–0.83), which was higher than covariates alone including individuals' age, gender, and smoking status (AUC = 0.56 ,95% CI = 0.52–0.60, *P* < 2 × 10^−16^ for a difference between the two AUCs). Including the covariates with mdNLR did not improve the ROC curve, and AUC remained at 0.80 (95% CI = 0.77–0.84) (Figure 3).

### 3.3. Elevated mdNLR Is Associated with Increased Odds of Being a Preclinical RA Case

We found increased odds of being a preclinical RA after adjusting for batch effects (OR_adj_ = 4.30, 95% CI = 1.52–21.71, *P* = 0.029; Table 2). We did not observe a difference in the mean mdNLR between preclinical RA case and controls (Table 1). There was no association between mdNLR in pregnancy and time to RA diagnosis (Supplementary Figure 3), and mdNLR had limited diagnostic ability to discriminate preclinical RA from controls in this small sample (Supplementary Figure 4(a)).

### 3.4. mdNLR Is Not Associated with Treated Rheumatoid Arthritis

Women with RA who were assumed to be using DMARDs had a lower mean mdNLR compared to controls (Table 1), but confidence intervals around the odds ratio crossed the null in multivariate model adjusted for batch (OR = 0.34, 95% CI = 0.05–1.82, *P* = 0.23; Table 2). In a ROC analysis, mdNLR was unable to discriminate RA cases from controls (Supplementary Figure 4(b)).

### 3.5. mdNLR Is Not Associated with TNFi Treatment Response

A higher mdNLR index was not associated with increased odds of being a poor responder to TNFi (etanercept) treatment compared to a good responder (OR = 1.10, 95% CI = 0.75–1.68, *P* = 0.64; Table 2).

## 4. Discussion

In this investigation of four datasets, we have identified mdNLR as a marker of SI and RA status. We were able to demonstrate the utility of mdNLR as a marker of chronic SI in untreated RA, which is in line with the findings of previous studies [12, 32]. The mdNLR had an improved diagnostic ability over age, sex, and smoking alone, although the poor performance of the covariates may be explained by the original study design, which matched cases and controls on smoking status. We also found associations between RA status and methylation at five myeloid differentiation CpGs, which may potentially indicate myeloid suppression [16]. This finding may reflect the presence of myeloid-derived suppressor cells (MDSCs) in RA cases. MDSCs are known to act as suppressors of antitumour immune responses [33, 34] and have been shown to play a role in arthritic progression in mice and RA patients [35]. Interestingly, we found that pregnant women with elevated mdNLR had increased odds of being a preclinical RA compared to disease-free pregnant women, suggesting that mdNLR may have some utility in identifying RA before clinical features manifest. However, it should be noted that prior to batch effect correction, there was no difference in the mean mdNLR between RA cases and controls suggesting that any difference between the two groups are potentially masked by batch effects. These findings would need replication in an independent dataset. Similarly, we found no association between mdNLR and time to diagnosis; this may be due to low statistical power in this small dataset.

We found lower (nonsignificant) mdNLR in treated RA cases compared to controls; these findings are not surprising because treatment with DMARDs is hypothesized to reduce inflammation [36] and would therefore affect mdNLR. For example, a recent meta-analysis showed that treatment with TNFi reduces SI [37]. TNF is a key cytokine in the inflammatory response, stimulating both its own production and the production of many other inflammatory cytokines. It has been shown to have a dominant role in RA, hence the rationale for TNF inhibition as a therapeutic target in RA [38]. We hypothesized that a high inflammation index at the baseline (reflected by a high mdNLR) may be associated with TNFi treatment response. However, our finding of no association between mdNLR and response to the TNFi etanercept suggests that response to TNFi may be predominantly a genetically and/or epigenetically driven process independent of the baseline SI. Although with the limited sample size, we are unable to conclusively prove the role of SI in TNFi treatment response.

Strengths of our study include the use of four datasets, giving us the ability to explore varied roles for mdNLR in untreated, treated, and preclinical RA cases, as well as in relation to treatment response. We carried out multivariate analyses adjusting for appropriate potential confounders and sources of variation. For example, smoking is suggested to play a critical role in the development of RA through altered immunologic function [1], and our recent study identified an altered number of immune cells in response to smoking [39]. The original study that generated the new onset RA dataset attempted to control potential confounding by smoking by matching cases and controls on smoking status [17]. In our analyses, we further adjusted models for smoking status to address any residual confounding that may have occurred due to smoking.

The limitations of our study are as follows. First, we were unable to identify independent datasets in which to replicate our findings. This could be attributed to the fact that most of the published and publicly available RA datasets have genome-wide DNA methylation data on specific cell types like T and B lymphocytes [40, 41] or fibroblast-like synoviocytes [42] which cannot be used to derive mdNLR. Second, in the new onset RA dataset, we were only able to adjust for the variables that were publicly available, which included age, gender, and smoking status. However, we believe that by using surrogate variables, we were able to capture and adjust the potential sources of heterogeneity that are not captured by variables included in the model. Third, we were unable to compare mdNLR to NLR due to the absence of directly measured blood cell type proportions for the studied datasets. Although, confidence in our measured methylation-derived SI is strengthened by previous studies that have validated the use of methylation-derived cell counts in estimating SI [16, 26]. Fourth, in the ALSPAC sample, we assumed that all prevalent RA cases were undergoing some form of treatment because they all reported that they had been diagnosed by a doctor; however, treatment and dosage data were not available. Finally, we were unable to adjust for the ACPA levels for the preclinical RA dataset as the ACPA levels were not measured for the samples in the ALSPAC dataset.

The NLR is routinely derived from absolute neutrophil and lymphocyte counts from a complete blood count in RA patients. We envisage that mdNLR will be useful for epidemiologists as a research tool to investigate SI using archival blood samples in the absence of cell-based NLR estimates. Our confidence in estimating mdNLR as a measure of systemic inflammation is strengthened by previous studies that have reported a high concordance between cell-based and methylation-derived NLR [16, 26].

## 5. Conclusions

In conclusion, we have demonstrated that mdNLR is elevated during RA disease onset, but not in prevalent cases. This may be reflective of a higher SI in RA patients prior to DMARD therapy. Our findings would be useful in estimating SI especially in prospective studies where the estimates of leukocyte subtypes were not recorded at recruitment. It remains to be tested if mdNLR measures SI independent of acute phase proteins such as CRP. In the future, it would be interesting to validate our findings in a large prospective study of RA and note how early we can detect SI during the disease development. Finally, we would be interested in testing if mdNLR may be a useful tool to evaluate SI in a chronic disease research setting, especially in large prospective studies with archived blood samples.

## Figures and Tables

**Table 1:** Sample characteristics of datasets.

Study	Characteristics	Reference group	Comparison group	*P* value
New onset RA (GSE42861) (*n* = 689) Reference: Liu et al. [17]		Controls (*n* = 335)	ACPA-associated cases (*n* = 354)	
	Mean mdNLR (SD)	2.02 (1.37)	4.67 (6.28)	<2×10–16^#^
	Mean age (range)	52.75 (20–70)	51.15 (18–69)	0.10^#^
	Gender			
	Males	96 (29%)	101 (29%)	0.99^@^
	Females	239 (71%)	253 (71%)	
	Smoking			
	Current	89 (27%)	111 (31%)	0.27^@^
	Former	108 (32%)	120 (34%)	
	Never	101 (30%)	92 (26%)	
	Occasional	35 (10%)	31 (9%)	
	NA	2 (1%)	0 (0%)	

Preclinical RA (ALSPAC sample during pregnancy) (*n* = 248) Reference: Relton et al. [20]		Controls (*n* = 200)	Cases (*n* = 48)	
	Mean mdNLR (SD)	2.78 (1.30)	2.65 (1.37)	0.55^#^
	Mean age (range)	29.32 (16–41)	29.30 (21–40)	0.79^#^
	Number of missing age	1	1	
	Smoking			
	Current	25 (13%)	7 (15%)	0.64^@^
	Former	49 (24%)	13 (27%)	
	Never	125 (62%)	27 (56%)	
	NA	1 (1%)	1 (2%)	
	Mean time to diagnosis in years (SD)	—	13.6 (4.8)	NA

Prevalent treated RA (ALSPAC 18 years after pregnancy) (*n* = 221) Reference: Relton et al. [20]		Controls (*n* = 200)	Cases (*n*=21)	
	Mean mdNLR (SD)	1.35 (0.68)	1.30 (0.41)	0.97^#^
	Mean age (range)	47.42 (35–59)	48.45 (40–58)	0.31^#^
	Number of missing age	21	1	
	Smoking			
	Current	11 (6%)	4 (19%)	0.11^@^
	Former	75 (37%)	8 (38%)	
	Never	111 (55%)	9 (43%)	
	NA	3 (2%)	0 (0%)	

TNFi response dataset (BRAGGSS) (*n* = 71) Reference: Plant et al. [21]		Good responders (*n* = 36)	Poor responders (*n* = 35)	
	Mean mdNLR (SD)	2.09 (1.15)	2.22 (1.84)	0.57^#^
	Mean age (range)	54.6 (28–78)	59.9 (36–87)	0.07^#^
	Gender			
	Males	8 (22%)	6 (17%)	0.77^@^
	Females	28 (78%)	29 (83%)	
	Smoking			
	Current	5 (14%)	4 (11%)	0.70^@^
	Former	14 (39%)	10 (29%)	
	Never	11 (31%)	12 (34%)	
	NA	6 (16%)	9 (26%)	
	Mean DAS28 score (SD)	5.7 (0.70)	5.5 (1.14)	0.28^#^
	Mean HAQ score (SD)	1.7 (0.52)	2.0 (0.59)	0.06^#^

^#^Wilcoxon rank sum test; ^@^Fisher's exact test or Chi-squared test.

**Table 2:** Association between mdNLR and rheumatoid arthritis status.

Study	Variables	OR (95% CI)	*P* value
New onset RA (GSE42861) (*n* = 685)^∗^			
	mdNLR	2.32 (1.95–2.80)	<2×10^−16^
	Age	1.00 (0.98–1.01)	0.81
	Gender		
	Female	Reference	NA
	Male	0.82 (0.52–1.28)	0.38
	Smoking status		
	Current	Reference	NA
	Former	0.90 (0.55–1.47)	0.67
	Occasional	0.92 (0.45–1.86)	0.81
	Never	0.68 (0.41–1.13)	0.14

Preclinical RA (ALSPAC sample during pregnancy) (*n* = 244)^@^			
	mdNLR	4.30 (1.52–21.71)	0.029
	Age	1.00 (0.79–1.25)	0.996
	Smoking status		
	Current	Reference	NA
	Former	0.03 (0.00–1.50)	0.105
	Never	0.07 (0.00–0.73)	0.043

Prevalent treated RA (ALSPAC 18 years after pregnancy) (*n* = 196)^@^			
	mdNLR	0.34 (0.05–1.82)	0.23
	Age	0.97 (0.82–1.15)	0.81
	Smoking status		
	Current	Reference	NA
	Former	0.19 (0.01-2.67)	0.23
	Never	0.15 (0.01–1.77)	0.15

TNFi response dataset (BRAGGSS) (*n* = 56)^@^			
	mdNLR	1.10 (0.75–1.68)	0.64
	Age	1.03 (0.98–1.09)	0.26
	Gender		
	Female	Reference	NA
	Male	0.60 (0.10–3.00)	0.54
	Smoking status		
	Current	Reference	NA
	Former	0.56 (0.10–3.18)	0.51
	Never	1.02 (0.19–5.69)	0.98
	DAS28	0.62 (0.28–1.25)	0.20
	Use of DMARDs	1.86 (0.31–15.15)	0.51

^∗^Model was in addition adjusted for 10 surrogate variables. ^@^Model was in addition adjusted for batch.

## Data Availability

ALSPAC data management policies do not permit datasets to be made publicly available due to data confidentiality and the potential to identify individual study participants from the data. Data used will be made available following an approved request from the ALSPAC executive (alspac-exec@bristol.ac.uk). The ALSPAC data management plan is available online: http://www.bristol.ac.uk/media-library/sites/alspac/documents/researchers/data-access/alspac-data-management-plan.pdf. BRAGGSS data is available on request from Professor Anne Barton (anne.barton@manchester.ac.uk) or is available from the corresponding author upon request.

## Supplementary Materials

Supplementary Materials Supplementary Table 1: mean methylation (beta values) of mdNLR-associated CpGs in new onset RA cases and controls. Supplementary Figure 1: correlation between two algorithms for estimating mdNLR. *x*-axis represents mdNLR estimated by minfi function (“estimateCellCounts”), and *y*-axis represents mdNLR estimated by IDOL algorithm. Supplementary Figure 2: comparison of the myeloid-associated CpGs between new onset RA cases and controls. *x*-axis indicates the myeloid differentiation, mdNLR-associated CpGs. *y*-axis indicates the DNA methylation levels (beta values) for each CpG site in new onset RA cases and controls. Supplementary Figure 3: relationship between mdNLR in preclinical RA cases at baseline (during pregnancy) and time to diagnosis of RA. Supplementary Figure 4: diagnostic ability of mdNLR to distinguish preclinical and prevalent treated RA cases and controls. Each ROC curve was generated from a different classifier: shown on the left hand top corner along with the area under the ROC curve (AUC) values for (a) preclinical and (b) prevalent treated RA cases and controls. Covariates included age and smoking status (all participants were female).

## References

[1] Firestein G. S., McInnes I. B. (2017). Immunopathogenesis of rheumatoid arthritis. *Immunity*.

[2] Wright H. L., Moots R. J., Edwards S. W. (2014). The multifactorial role of neutrophils in rheumatoid arthritis. *Nature Reviews Rheumatology*.

[3] Kaplan M. J. (2013). Role of neutrophils in systemic autoimmune diseases. *Arthritis Research & Therapy*.

[4] Jaeger B. N., Donadieu J., Cognet C. (2012). Neutrophil depletion impairs natural killer cell maturation, function, and homeostasis. *The Journal of Experimental Medicine*.

[5] Willems J. M., Trompet S., Blauw G. J., Westendorp R. G. J., de Craen A. J. M. (2010). White blood cell count and C-reactive protein are independent predictors of mortality in the oldest old. *The Journals of Gerontology Series A, Biological Sciences and Medical Sciences*.

[6] Kisacik B., Tufan A., Kalyoncu U. (2008). Mean platelet volume (MPV) as an inflammatory marker in ankylosing spondylitis and rheumatoid arthritis. *Joint, Bone, Spine*.

[7] Roxburgh C. S. D., McMillan D. C. (2014). Cancer and systemic inflammation: treat the tumour and treat the host. *British Journal of Cancer*.

[8] Verdoia M., Barbieri L., di Giovine G. (2016). Neutrophil to lymphocyte ratio and the extent of coronary artery disease: results from a large cohort study. *Angiology*.

[9] Kim S., Eliot M., Koestler D. C., Wu W. C., Kelsey K. T. (2018). Association of neutrophil-to-lymphocyte ratio with mortality and cardiovascular disease in the Jackson Heart Study and modification by the Duffy antigen variant. *JAMA Cardiology*.

[10] Templeton A. J., McNamara M. G., Šeruga B. (2014). Prognostic role of neutrophil-to-lymphocyte ratio in solid tumors: a systematic review and meta-analysis. *Journal of the National Cancer Institute*.

[11] Pan L., Du J., Li T., Liao H. (2017). Platelet-to-lymphocyte ratio and neutrophil-to-lymphocyte ratio associated with disease activity in patients with Takayasu’s arteritis: a case-control study. *BMJ Open*.

[12] Uslu A. U., Kucuk A., Sahin A. (2015). Two new inflammatory markers associated with disease activity score-28 in patients with rheumatoid arthritis: neutrophil-lymphocyte ratio and platelet-lymphocyte ratio. *International Journal of Rheumatic Diseases*.

[13] Ghang B., Kwon O., Hong S., Lee C. K., Yoo B., Kim Y. G. (2017). Neutrophil-to-lymphocyte ratio is a reliable marker of treatment response in rheumatoid arthritis patients during tocilizumab therapy. *Modern Rheumatology*.

[14] Titus A. J., Gallimore R. M., Salas L. A., Christensen B. C. (2017). Cell-type deconvolution from DNA methylation: a review of recent applications. *Human Molecular Genetics*.

[15] Koestler D. C., Usset J., Christensen B. C. (2017). DNA methylation-derived neutrophil-to-lymphocyte ratio: an epigenetic tool to explore cancer inflammation and outcomes. *Cancer Epidemiology, Biomarkers & Prevention*.

[16] Wiencke J. K., Koestler D. C., Salas L. A. (2017). Immunomethylomic approach to explore the blood neutrophil lymphocyte ratio (NLR) in glioma survival. *Clinical Epigenetics*.

[17] Liu Y., Aryee M. J., Padyukov L. (2013). Epigenome-wide association data implicate DNA methylation as an intermediary of genetic risk in rheumatoid arthritis. *Nature Biotechnology*.

[18] Padyukov L., Silva C., Stolt P., Alfredsson L., Klareskog L. (2004). A gene-environment interaction between smoking and shared epitope genes in HLA-DR provides a high risk of seropositive rheumatoid arthritis. *Arthritis &Rheumatism*.

[19] Klareskog L., Stolt P., Lundberg K. (2006). A new model for an etiology of rheumatoid arthritis: smoking may trigger HLA-DR (shared epitope)-restricted immune reactions to autoantigens modified by citrullination. *Arthritis and Rheumatism*.

[20] Relton C. L., Gaunt T., McArdle W. (2015). Data resource profile: Accessible Resource for Integrated Epigenomic Studies (ARIES). *International Journal of Epidemiology*.

[21] Plant D., Webster A., Nair N. (2016). Differential methylation as a biomarker of response to etanercept in patients with rheumatoid arthritis. *Arthritis & Rhematology*.

[22] Maksimovic J., Gordon L., Oshlack A. (2012). SWAN: subset-quantile within array normalization for Illumina Infinium HumanMethylation450 BeadChips. *Genome Biology*.

[23] Chen Y. A., Lemire M., Choufani S. (2013). Discovery of cross-reactive probes and polymorphic CpGs in the Illumina Infinium HumanMethylation450 microarray. *Epigenetics*.

[24] Aryee M. J., Jaffe A. E., Corrada-Bravo H. (2014). Minfi: a flexible and comprehensive Bioconductor package for the analysis of Infinium DNA methylation microarrays. *Bioinformatics*.

[25] Houseman E. A., Accomando W. P., Koestler D. C. (2012). DNA methylation arrays as surrogate measures of cell mixture distribution. *BMC Bioinformatics*.

[26] Koestler D. C., Jones M. J., Usset J. (2016). Improving cell mixture deconvolution by identifying optimal DNA methylation libraries (IDOL). *BMC Bioinformatics*.

[27] Leek J. T., Johnson W. E., Parker H. S., Jaffe A. E., Storey J. D. (2012). The sva package for removing batch effects and other unwanted variation in high-throughput experiments. *Bioinformatics*.

[28] McGregor K., Bernatsky S., Colmegna I. (2016). An evaluation of methods correcting for cell-type heterogeneity in DNA methylation studies. *Genome Biology*.

[29] Blagojevic M., Jinks C., Jeffery A., Jordan K. P. (2010). Risk factors for onset of osteoarthritis of the knee in older adults: a systematic review and meta-analysis. *Osteoarthritis and Cartilage*.

[30] Sugiyama D., Nishimura K., Tamaki K. (2010). Impact of smoking as a risk factor for developing rheumatoid arthritis: a meta-analysis of observational studies. *Annals of the Rheumatic Diseases*.

[31] Robin X., Turck N., Hainard A. (2011). pROC: an open-source package for R and S+ to analyze and compare ROC curves. *BMC Bioinformatics*.

[32] Mercan R., Bitik B., Tufan A. (2016). The association between neutrophil/lymphocyte ratio and disease activity in rheumatoid arthritis and ankylosing spondylitis. *Journal of Clinical Laboratory Analysis*.

[33] Marvel D., Gabrilovich D. I. (2015). Myeloid-derived suppressor cells in the tumor microenvironment: expect the unexpected. *The Journal of Clinical Investigation*.

[34] Lindau D., Gielen P., Kroesen M., Wesseling P., Adema G. J. (2013). The immunosuppressive tumour network: myeloid-derived suppressor cells, regulatory T cells and natural killer T cells. *Immunology*.

[35] Guo C., Hu F., Yi H. (2016). Myeloid-derived suppressor cells have a proinflammatory role in the pathogenesis of autoimmune arthritis. *Annals of the Rheumatic Diseases*.

[36] Choy E., Ganeshalingam K., Semb A. G., Szekanecz Z., Nurmohamed M. (2014). Cardiovascular risk in rheumatoid arthritis: recent advances in the understanding of the pivotal role of inflammation, risk predictors and the impact of treatment. *Rheumatology (Oxford)*.

[37] Roubille C., Richer V., Starnino T. (2015). The effects of tumour necrosis factor inhibitors, methotrexate, non-steroidal anti-inflammatory drugs and corticosteroids on cardiovascular events in rheumatoid arthritis, psoriasis and psoriatic arthritis: a systematic review and meta-analysis. *Annals of the Rheumatic Diseases*.

[38] Monaco C., Nanchahal J., Taylor P., Feldmann M. (2015). Anti-TNF therapy: past, present and future. *International Immunology*.

[39] Ambatipudi S., Cuenin C., Hernandez-Vargas H. (2016). Tobacco smoking-associated genome-wide DNA methylation changes in the EPIC study. *Epigenomics*.

[40] Glossop J. R., Glossop J. R., Emes R. D. (2014). Genome-wide DNA methylation profiling in rheumatoid arthritis identifies disease-associated methylation changes that are distinct to individual T- and B-lymphocyte populations. *Epigenetics*.

[41] Glossop J. R., Emes R. D., Nixon N. B. (2016). Genome-wide profiling in treatment-naive early rheumatoid arthritis reveals DNA methylome changes in T and B lymphocytes. *Epigenomics*.

[42] Nakano K., Whitaker J. W., Boyle D. L., Wang W., Firestein G. S. (2013). DNA methylome signature in rheumatoid arthritis. *Annals of the Rheumatic Diseases*.

