# Supplementary material for: Assessing the Role of DNA Methylation-Derived Neutrophil-to-Lymphocyte Ratio in Rheumatoid Arthritis
Source: J Immunol Res. 2018 Aug 14;2018:2624981. doi: 10.1155/2018/2624981 (PMC6112073; doi:10.1155/2018/2624981)

Supplementary Material

Supplementary Table 1

Myeloid differentiation CpGs	Mean controls (range)	Mean RA cases (range)	*P-value*[#]
cg00901982	0.37 (0.15-0.60)	0.29 (0.09-0.47)	$< 2.2x10^{-16}$
cg25938803	0.43 (0.23-0.76)	0.34 (0.13-0.66)	$< 2.2x10^{-16}$
cg01591037	0.43 (0.19-0.81)	0.34 (0.12-0.59)	$< 2.2x10^{-16}$
cg03621504	0.33 (0.15-0.53)	0.26 (0.09-0.47)	$< 2.2x10^{-16}$
cg10456459	0.48 (0.20-0.84)	0.38 (0.14-0.62)	$< 2.2x10^{-16}$

[#]Wilcoxon rank sum test

Supplementary Figure 1

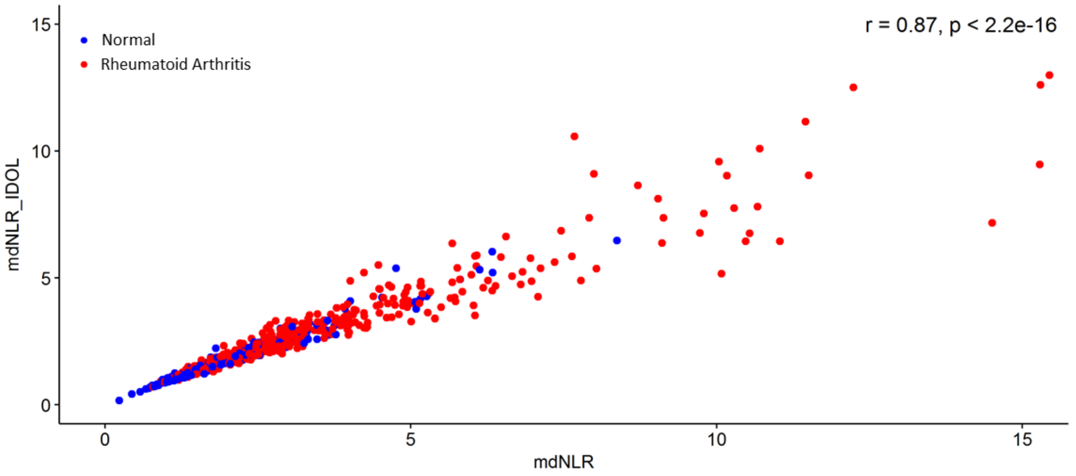

Supplementary Figure 2

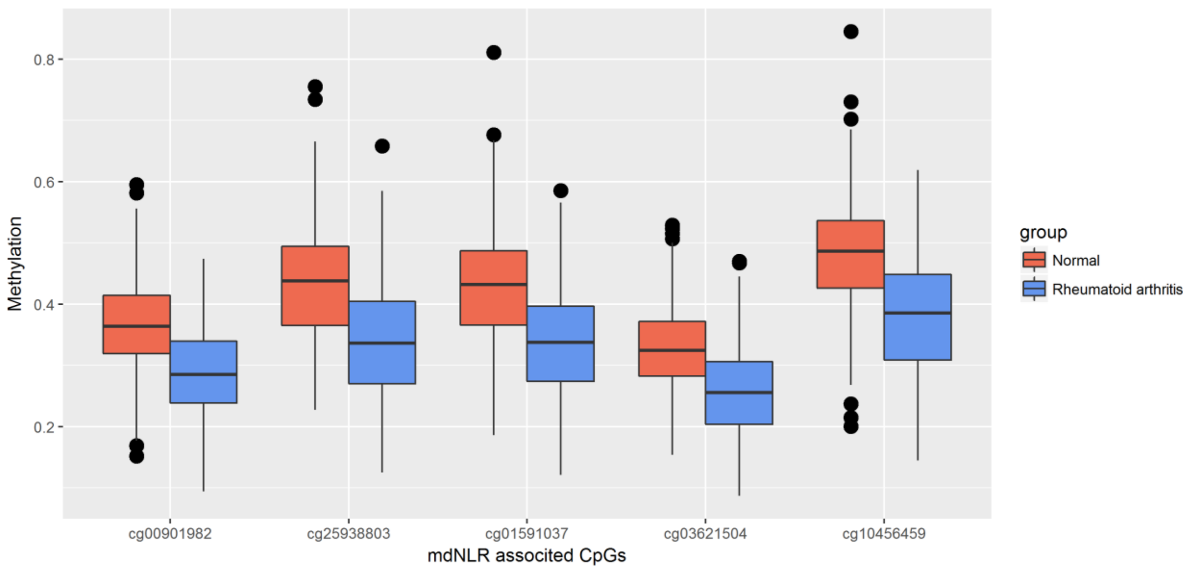

Supplementary Figure 3

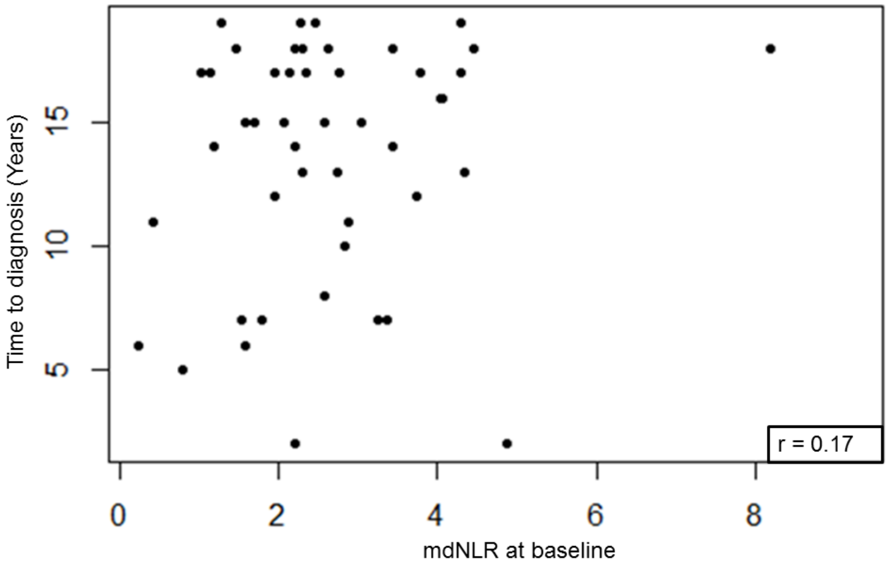

Supplementary Figure 4

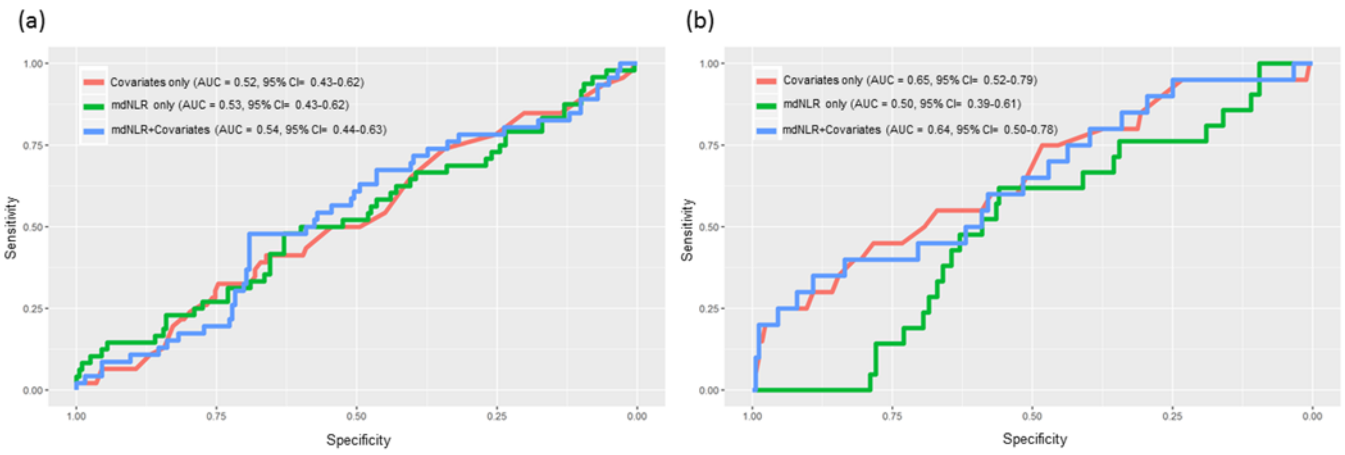

Supplement: Supplementary Materials — Supplementary Table 1: mean methylation (beta values) of mdNLR-associated CpGs in new onset RA cases and controls. Supplementary Figure 1: correlation between two algorithms for estimating mdNLR. x-axis represents mdNLR estimated by minfi function (“estimateCellCounts”), and y-axis represents mdNLR estimated by IDOL algorithm. Supplementary Figure 2: comparison of the myeloid-associated CpGs between new onset RA cases and controls. x-axis indicates the myeloid differentiation, mdNLR-associated CpGs. y-axis indicates the DNA methylation levels (beta values) for each CpG site in new onset RA cases and controls. Supplementary Figure 3: relationship between mdNLR in preclinical RA cases at baseline (during pregnancy) and time to diagnosis of RA. Supplementary Figure 4: diagnostic ability of mdNLR to distinguish preclinical and prevalent treated RA cases and controls. Each ROC curve was generated from a different classifier: shown on the left hand top corner along with the area under the ROC curve (AUC) values for (a) preclinical and (b) prevalent treated RA cases and controls. Covariates included age and smoking status (all participants were female). [file 2624981.f1.pdf]
